# Fecal Microbiota and Human Intestinal Fluid Transplantation: Methodologies and Outlook

**DOI:** 10.3389/fmed.2022.830004

**Published:** 2022-05-18

**Authors:** Ye Chen, Lin Zhiliang, Cui Jiaqu, Lv Xiaoqiong, Zhang Shaoyi, Ma Chunlian, Yan Yinmei, Yang Bo, Zhao Di, Tian Hongliang, Li Ning, Chen Qiyi, Qin Huanlong

**Affiliations:** Department of Colorectal Disease Specialty, Clinical Research Center for Digestive Diseases, The Tenth People's Hospital, Tongji University, Shanghai, China

**Keywords:** human intestinal fluid transplantation, fecal microbiota transplantation, gut microbial therapeutics, methodologies, donor

## Abstract

Fecal microbiota transplantation (FMT) is a therapy that involves the transplantation of healthy human fecal microorganisms into the gut of patients to rebuild or consolidate the intestinal microecology. It has been utilized in many diseases. However, FMT had a limited effect on patients with small intestinal diseases because of the unique ecological characteristics of the microorganisms. Thus, we proposed a new microecology transplantation therapy called human intestinal fluid transplantation (HIFT). Human intestinal fluid can be collected through a nasojejunal tube and be made into capsules using the freeze-dried powder method. In addition, strict standards for donor screening and management have been established. We are currently developing a high-standard HIFT preparation system and conducting high-quality clinical studies to validate the safety and efficacy of HIFT combined with FMT.

## Introduction

Fecal microbiota transplantation (FMT) is a new therapy that involves the transplantation of healthy human fecal microorganisms into the gut of patients to rebuild or consolidate the intestinal microecology (1). Many diseases have been found to be associated with intestinal flora disturbance, including intestinal diseases, such as functional bowel disease (2), inflammatory bowel disease (3), and infectious diarrhea (4); and extra-intestinal diseases, such as Parkinson's disease (5), autism spectrum disorder (ASD) (6), and amyotrophic lateral sclerosis (7). Many studies have showed that the microbial community structure and functions could be normalized after FMT in human, instead of destroying the original structure (2–4, 6). However, FMT is not a panacea for all kinds of diseases, especially in diseases associated with the brain-gut axis and small intestinal bacterial overgrowth (8).

Given that FMT mainly treats the colon flora, the 4-meter-long small intestine is not given the priority it deserves. Small intestine is one of the most important organs in human beings, which has lots of digestive enzymes, microorganisms, immunoglobulins and other vital substances (9–11). It is involved in nutrient absorption, secretion, metabolism and immune functions. However, few interventions are used in small intestinal diseases. Recent studies have also confirmed that bacteria from different segments of the gut colonize homologous segments after transplantation. These microorganisms have unique ecological characteristics (12), and the application of 16S rRNA technology has brought a new understanding to the study of microorganisms. It was reported that there were amounts of bacteria in the stomach and small intestine, where it was previously thought that there was only few (13, 14). In clinical practice, human intestinal fluids (HIF) reinfusion can significantly improve intestinal function in patients with severe intestinal dysfunction (15, 16). Homologous HIF may have better tolerance than enteral nutrient solution because of the living substance. Therefore, human intestinal fluid transplantation (HIFT) from the healthy population may be more effective for patients with intestinal function disturbances than FMT. But there is no effective method to collect human intestinal fluid in clinic. In this study, we summarize the first establishment of standardized HIFT preparation, which is used in the treatment of intestinal dysfunction diseases.

## Donor Selection and Management

HIFT is a new therapy without a standardized methodology yet. To ensure the safety and efficacy of HIFT (17–19), the criteria for HIFT donor selection and management were based on the China expert consensus on the establishment of standardized methodology and clinical application for FMT (20). The strict donor screening criteria included objective criteria, psychological evaluation, and history of diseases, which fully evaluated the past and recent potentially harmful behaviors and the risks for infection transmission:

**Donor screening criteria and management criteria:**

- **Objective criteria**
  – Age 18–30, male or female, body mass index of 18.5–22.9 kg/m^2^.
  – Normr negative hematology tests: blood routine test, hepatic and renal function, electrolytes and c-reactive protein, infectious hepatitis, HIV, syphilis, Epstein-Barr virus, cytomegalovirus, nematode, amoeba, and other pathogens.
  – Normal or negative stool tests: feces regular test, occult blood test, *Clostridium Difficile, Campylobacteria, Salmonella, Shigella, Shiga-toxigenic Escherichia coli*, worm eggs, vesicles, parasites, spores, norovirus, rotavirus, and multiple drug resistance genes (such as carbapenem-resistant enterobacteriaceae, extended-spectrum β-lactamase-producing bacteria, methicillin-resistant staphylococcus aureus and other drug-resistant bacteria).
- **Psychological evaluation**
  – Assessed as having a good psychological state by a cardiologist or psychological consultant.
  – Normal scores in Self-rating Depression Scale, Self-rating Anxiety Scale, and Pittsburgh Sleep Quality Index.
- **History of diseases**
  – Past History: without gastrointestinal symptoms in recent 2 weeks, no antibiotics, acid inhibitors, immunosuppressants, or chemotherapeutic drug use in the last 3 months, no chronic pain symptoms, no history of digestive surgery, no history of infection and infectious disease exposure, no allergic disease, no autoimmune disease, no metabolic disease, no cardiovascular and cerebrovascular diseases, no neuropathy, no psychosis, no malignancy, no growth hormone, insulin, coagulation factors, or other injection treatment.
  – Personal history: have a regular routine, healthy diet and harmonious family, and no bad sexual history; no smoking, drinking, and drug addiction history; no vaccination, drug trial, skin damage, and contact with tropical areas in the last 6 months.
  – Family history: no family history of gastrointestinal diseases, malignant tumors, or infectious diseases.
  – Others: not pregnant, not in menstrual period.
- **Archive and follow-up system establishment**
  – Standard donor file establishment, including recording each inspection result, HIF donation, and related treatment.
  – Follow-up system establishment, to ensure that the donors regularly complete and pass the physical examination and donation requirements.
- **Donor management group establishment**
  – The donor management should be in charge of the full-time donor managers of the HIFT center, including at least 1 principal and 2 assistants.
  – The donor managers should maintain regular communication with the donors, establish a good trust relationship, and carry out necessary management and intervention on the donor's lifestyle and diet structure. They should immediately correct the unhealthy lifestyle and diet structure, and eliminate unqualified donors according to the follow-up results.
- **Informed consent of the donors**
  – Donor candidates should be fully informed and signed the informed consent before screening.
  – The HIFT donor should be fully informed and signed the informed consent for nasointestinal tube catheterization before donation.
- **HIFT Donor donation requirements**
  – The donors shall ensure the continuity of the donation. Each donation can be made for 3–7 consecutive days, once every 1–2 months.
  – The amount of HIF donation should be no <350 mL per day after filtering, and the color of the HIF must be golden yellow.

The above items should be reviewed every 2 months. Only ~2% of the population can be screened as ideal donors. After screening, HIFT donors provided informed consent for the donation and nasointestinal tube catheterization during the HIF collection period. Every donor should obey the criteria of donor management to ensure the stabilization and safety of HIF. Stool and HIF samples of each donor were saved for 16s rRNA sequencing and composition analysis to ensure the basic stability of the bacterial community and biological components, and to allow tracing when recipients have adverse effects. In addition, some donors should restrict certain types of food for 5 days prior to the donation of HIF when recipients have food allergy or food intolerance symptoms.

## Preparation Methods of HIFT Capsules

**Figure 1:**
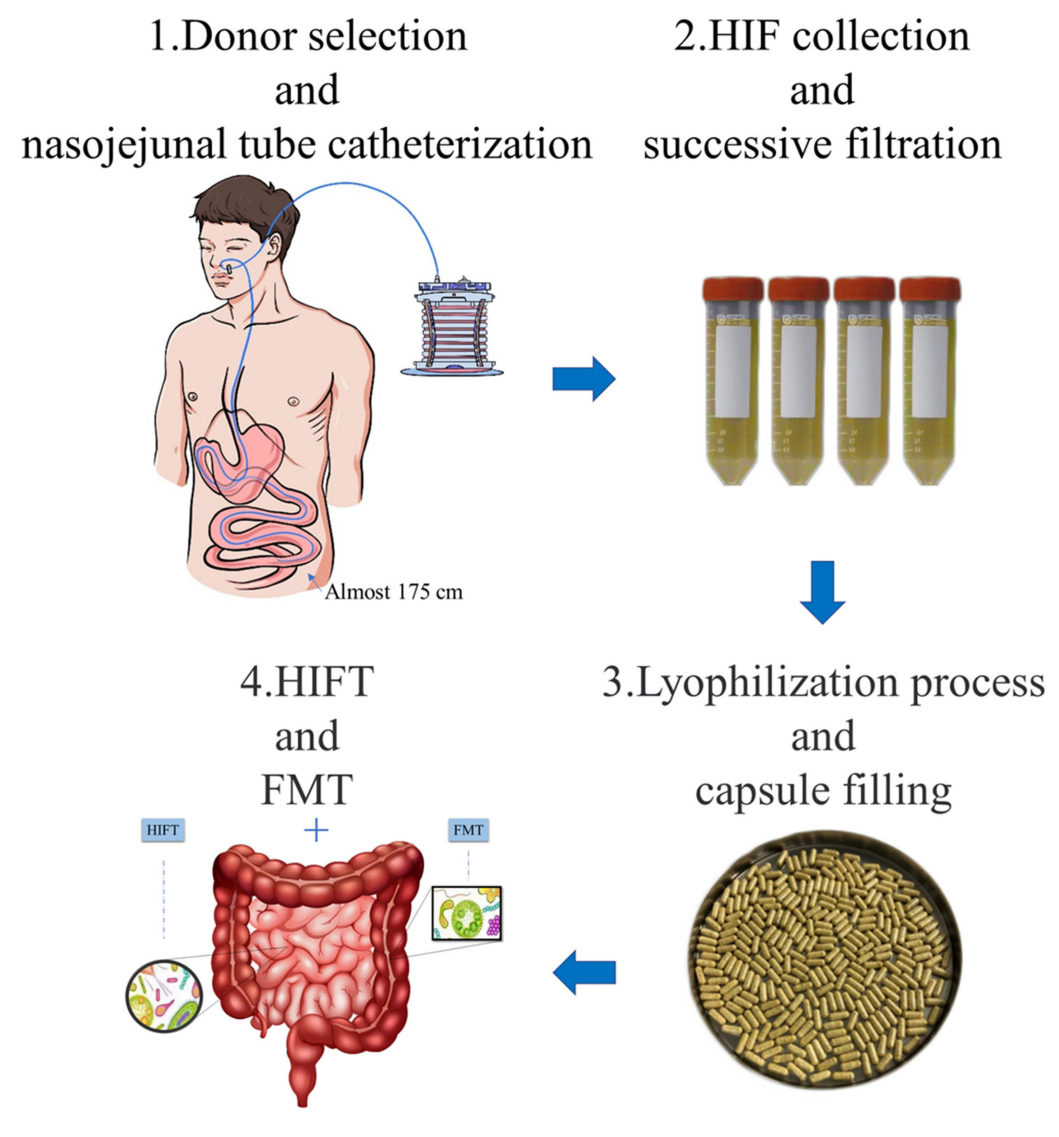
Preparation and application of HIFT capsules. 1. Donor selection and nasojejunal tube catheterization: eligible donors underwent nasojejunal tube catheterization by using a modified Flocare nasogastric feeding tube. Modified disposable sterile negative pressure collecting devices were connected to the catheter for continuous drainage. 2. HIF collection and successive filtration: the HIF was collected in the devices and transferred to filtration in 2 h. The filtration used screen cloths of 2.0 mm, 1.0 mm, and 0.5 mm. 3. Lyophilization process and capsule filling: 10% glycerin was added to the filtrate as a cryoprotectant and HIF was turned into the lyophilized powder by lyophilizer. The lyophilized powder was packaged in an enteric capsule shell of an acid-resistant acrylic resin, which would only disintegrate in small intestine. 4. HIFT and FMT: HIFT can be used in combination with FMT, in diseases that respond poorly to FMT treatment. In other words, it is a treatment of whole intestinal microbiota transplantation.

Donors underwent nasojejunal tube catheterization by using a modified Flocare nasogastric feeding tube, with a depth of 175 cm away from the nose. The modified disposable sterile negative pressure collecting devices were connected to the catheter for continuous drainage, which was controlled below 6.7 kPa. The collecting devices were changed every 2 h because the HIF may be metamorphic outside. After collection, HIF was successively filtered through screen cloths of 2.0, 1.0, and 0.5 mm. Then, 10% glycerin was added to the filtrate as a cryoprotectant and HIF was turned into the lyophilized powder by lyophilizer (19, 21), keeping the water content below 5% and mucus content below 10%. Finally, the lyophilized powder was packaged in an enteric capsule shell of an acid-resistant acrylic resin, which would only disintegrate in small intestine. The packaged capsules were stored in the −80°C refrigerator, and these were valid for 6 months (Figure 1).

The entire preparation process required information registration, HIF identification, weighing, and testing. A 2 mL HIF sample from each donation must be set aside for at least 6 months to allow tracing in case of adverse events. Donor information code, donation date, production date, expiration date, dose, and storage temperature were recorded.

## Indications and Contraindications

Although the efficiency of FMT for rCDI is 90% (22, 23), the response rates for other diseases do not have the same results. HIFT compensates for the loss of small intestinal microorganisms in the FMT. Thus, HIFT can be used in combination with FMT, in diseases that respond poorly to FMT treatment in preliminary, including digestive diseases (small intestinal bacterial overgrowth, inflammatory bowel disease, and obstructive functional constipation), neuropsychiatric disorders (ASD, anxious depression, and Parkinson's disease), metabolic disorders (diabetes, obesity, fatty liver, and hyperlipidemia), and immune systemic diseases (tumor immunity, allergic diseases, and chronic fatigue syndrome). The combination treatment, in other words, is a treatment of whole intestinal microbiota transplantation. However, the microorganism itself is an antigen. Bacterial translocation may be an important part of the exacerbation of the inflammatory response in the system (24). Abuse of FMT or HIFT can cause serious complications, including sepsis and death. Patients with congenital or acquired immune deficiency, recently received high-risk immunosuppressive or cytotoxic drugs, or with severely damaged intestinal mucosa, must not receive FMT or HIFT.

## Treatment of HIFT

Previous studies have suggested that the number of bacteria in the proximal small intestine is <10^5^ cfu/ml. 1 cfu is almost several numbers of bacteria. However, with the application of 16S rRNA and Trypan Blue test, the number of bacteria recorded in HIF is >3.0 ×10^8^/ml. There is a significant difference between these two values, and many new microorganisms have been identified (25). According to the test results, the following requirements were drawn. The amounts of viable bacteria were used as the standard therapeutic dose, which in the HIFT liquid should be ≥5.0 ×10^8^/ml, with the viable bacteria proportion ≥83%. The amounts of viable bacteria in the HIFT powder should be ≥2.0 ×10^6^/g and the viable bacteria proportion should be ≥81%. For adults, the therapeutic dose of HIFT liquid was 50 ml at each time, and for children, each dose was 1 ml/kg. Based on current technologies, the route for HIFT is only upper gastrointestinal tract, including nasojejunal tube, endoscopy and oral administration capsule. And, the nasojejunal tube and oral administration capsule can be repeated dosed. The HIFT treatment courses were consistent with the FMT. A standard course of HIFT was administered once daily for 6 consecutive days. Treatment is one course per month for at least two consecutive treatments. To mitigate this possible interaction, FMT was first followed by HIFT therapy. The interval between the two should be more than half an hour.

## Management of Adverse Effects

Although FMT has a low incidence of adverse effects (AEs) and its management varies from country to country, the risks of its clinical use must be carefully considered (24). The most common symptoms are nausea, emesis, abdominal distension, diarrhea, allergy, and fever. Most AEs are mild to moderate and are always self-limited (2). In fact, HIF contains fewer microorganisms and may be safer. Although HIF has not been thoroughly investigated, it can be considered as one of the most vital body fluids. Similarly, succus entericus reinfusion is an important therapy for treating severe patients with complex intestinal fistula, which can stabilize the intestinal mucosal barrier function and promote the recovery of intestinal function (15). It has been reported that autologous or allogeneic succus entericus reinfusion is safer and plays an extremely important role in the treatment of critically ill patients, and its effect was found to be even better than that of enteral nutrition alone (26, 27). The HIFT prevention and management of AEs were as follows: (1) Establishment of an AE reporting system; (2) Strict criteria on the indications and contraindications, and assessment of the risk of complications before HIFT; (3) Mild symptoms: continuous observation of clinical symptoms, such as mild dizziness, nausea, and gastrointestinal discomfort; (4) Moderate symptoms: symptomatic treatment and suspension of HIFT if necessary, such as oral antidiarrheal for diarrhea, oral non-steroidal anti-inflammatory drugs for fever, and intramuscular injection of metoclopramide for nausea and vomiting; (5) Severe symptoms: emergency treatment and termination of HIFT. Blood tests and symptomatic treatments are urgently needed. If enterogenic infection is suspected, blood culture tests should be conducted, and intravenous anti-infection or selective digestive decontamination should be administered (28). In addition, the patient's fecal pathogens and the donor's fluid and/or powder should be evaluated. Last but not least, HIFT should be applied and managed through the ethic committee and the local government. Though the regulatory issues of FMT vary from country to country, the goal of treatment is the same that every step of HIFT should be recorded and tested, to ensure that the bacteria are eligible and effective.

## Challenges and Future Considerations

The whole intestinal microbiota is a complex consortium with many components that have never been totally characterized in FMT. Likewise, the microbiota construction in HIF is even less known. Previous studies have tested the pH, bile salts, phospholipids, cholesterol, free fatty acids, pancreatic lipase, and other life active substance in HIF (29). It is used to know that due to the presence of gastric acid, bile acid, immunoglobulin IgA, and other bactericidal and antibacterial substances in the upper digestive tract (10), the small intestinal microbiota is minimal, which is <10^5^ cfu/ml (30, 31). This may be related to the innovation of microbiome detection technology and the discovery of many new microbiomes (13). Given that there are still numerous undiscovered bacteria, HIF may have more bacteria per unit volume. Nowadays, FMT has been proved as a therapy which has high security (1, 3, 8), though the knowledge has not been available enough either regarding the influence of transplanting the microbiota from person to person. Based on the standard treatment of FMT, HIFT is a brand-new treatment concept which may be safe. To ensure the safety, it is still important to carry out standard methodologies of HIFT, including donor/recipient screening, HIF preparation, route of transplantation, and informed consent.

The current preparation method of HIF is in a primary stage. It is the first time to achieve mass production. A variety of techniques have been used to achieve minimally invasive or non-invasive HIF biopsy in healthy people, including gastroscopy, capsule endoscopy, and nasointestinal tube insertion (32–34) (Table 1). Endoscopy is often limited by the limited depth of placement (32), and it is difficult to collect HIF in the fasting state (35). A new generation of capsule endoscope, called magnetically controlled sampling capsule endoscope, can collect 0.2–0.4 ml HIF through a negative pressure system (33). This yield cannot meet the treatment needs and the cost of this procedure is high (37). A double-lumen catheter, one kind of nasojejunal tubes, was mainly evaluated the physicochemical properties of HIF and the dissolution of drugs in the upper digestive tract (29, 36). The position of the catheter was proximal to the duodenum and distal to the jejunum, which needed the application of fluoroscopy (36). The tolerance of double-lumen catheter is poor, and in fact, the duodenum part is needless. The modified nasojejunal tube can be blindly placed into the distal jejunum in our center. It is non-invasive and simple, and the operating time of tube just need 2–5 min. The HIF should be continuously and repeatedly drained, and this preparation may be more optimized in the future.

**Table 1:** Different methods in achieving HIF biopsy in recent 5 years.

**References**	**Methods**	**Position**	**Recipients**
Leite et al. (32)	Duodenoscopy	Duodenum	Human
Tziatzios et al. (35)	Gastroscopy	Duodenum	Human
Ding et al. (33)	Magnetically controlled sampling capsule endoscope	Jejunum and ileum	Pigs
Riethorst et al. (29)	Double-lumen catheter	Duodenum and jejunum	Human
de la Cruz-Moreno et al. (36)	Double-lumen catheter	Duodenum and jejunum	Human
Riethorst et al. (34)	Double-lumen catheter	Duodenum and jejunum	Human and simulated intestinal fluids

It is certain that fecal therapy, no matter FMT or HIFT, will continue to be refined in methodologies and treatment concepts. According to the characteristics of microbiota distribution and functions of life active substances, HIFT may compensate the shortcomings of FMT. Thus, the whole intestinal microbiota transplantation can consist of FMT and HIFT, which may have greater impact on diseases. Although challenges exist, we will further analyze the components of HIF and conduct high-quality clinical studies to validate the safety and efficacy of HIFT combined with FMT.

## Data Availability Statement

The original contributions presented in the study are included in the article/Supplementary Material, further inquiries can be directed to the corresponding author.

## Ethics Statement

The studies involving human participants were reviewed and approved by Shanghai Tenth People's Hospital Ethics Committee. Written informed consent to participate in this study was provided by the participants or their legal guardian/next of kin.

## Author Contributions

CQ, LN, and QH made an investigation plan. CQ and YC prepared the draft of the manuscript. YC, YY, LX, and MC carried on the research work and provided data. YB, ZD, LZ, TH, and CJ manage donors and recipients. ZS, LZ, and CJ proofread and revised the manuscript. All authors agree to be accountable for the content of the work. All authors contributed to the article and approved the submitted version.

## Funding

This work was supported by Clinical Research Plan of SHDC (Nos. SHDC2020CR1030B and SHDC2020CR4026) and Shanghai Action Plan on Science, Technology and Innovation (21Y11908300).

## Conflict of Interest

The authors declare that the research was conducted in the absence of any commercial or financial relationships that could be construed as a potential conflict of interest.

## Publisher's Note

All claims expressed in this article are solely those of the authors and do not necessarily represent those of their affiliated organizations, or those of the publisher, the editors and the reviewers. Any product that may be evaluated in this article, or claim that may be made by its manufacturer, is not guaranteed or endorsed by the publisher.
