# Supplementary material for: Fecal Microbiota and Human Intestinal Fluid Transplantation: Methodologies and Outlook
Source: Front Med (Lausanne). 2022 May 18;9:830004. doi: 10.3389/fmed.2022.830004 (PMC9158325; doi:10.3389/fmed.2022.830004)
Supplement: Supplementary file 1 [file Data_Sheet_1.pdf]

From January to June, 2021, 11 HIFT donors were recruited in Shanghai Tenth People's Hospital. The clinical trial registration number is ChiCTR2100043929. All donors had signed informed consents. From the preliminary statistics before and after 1 course of treatment, the results showed that WIMT improved core symptoms in ASD patients (Autism Behavior Scale: P=0.0016; Childhood Autism Rating Scale: P=0.008). (Suppl. Figure) None of the 11 patients had serious AE, indicating the safety. Final results of the study will be published after the study completed. To our surprise, the amounts of bacteria in HIF were all more than 5.0×108cfu/ml, which was much higher than previous literature. Previous studies believed that, due to the presence of gastric acid, bile acid, immunoglobulin IgA and other bactericidal and antibacterial substances in the upper digestive tract, small intestinal microbiota was minimal, which was less than 105cfu/ml. This may be related to the innovation of microbiome detection technology and the large number of new microbiomes being discovered.

Suppl. Figure The effects of HIFT combined with FMT in ASD (A, B).

ABC: Autism Behavior Scale. CARS: Childhood Autism Rating Scale. SPSS25.0 software was used for statistical analysis. Data were tested for kolmogorov-Smirnov normality and pair T test. $P<0.05$ was considered statistically significant.

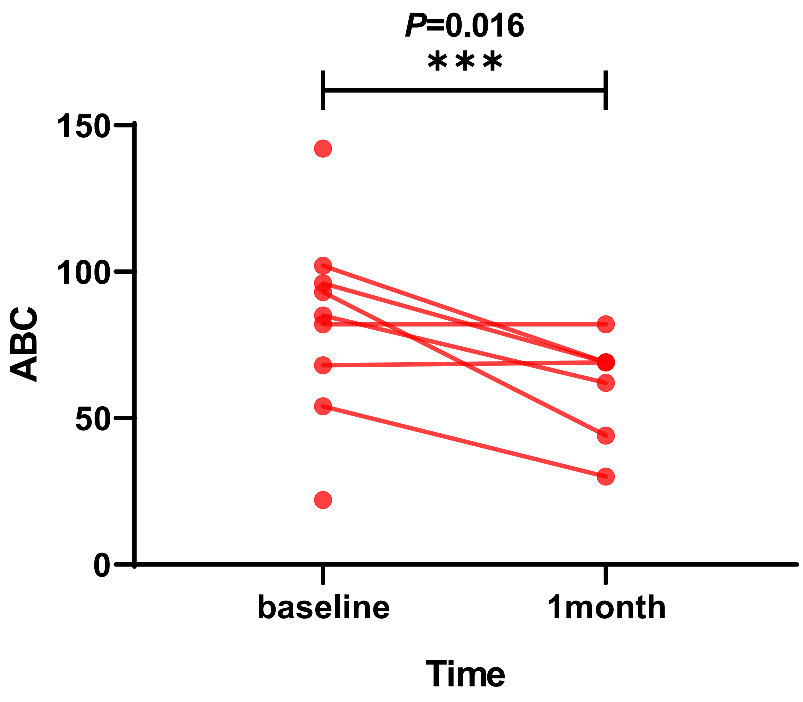

A.

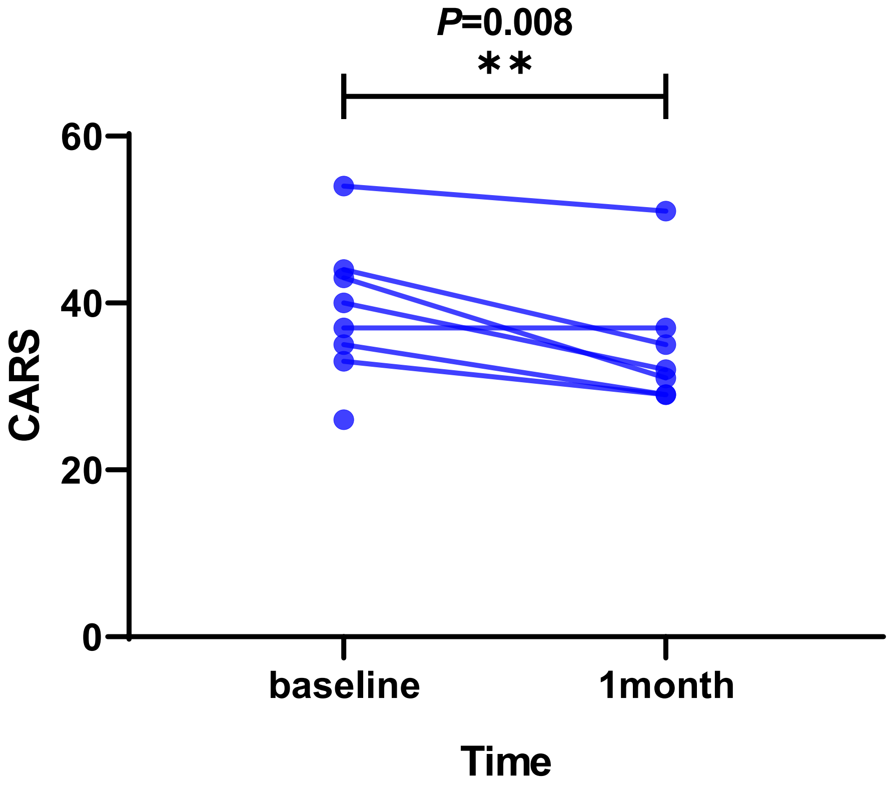

B.
